# Serial Combination of Toxic and Ischemic Renal Damages Causes Subsequent Chronic, Irreversible, and Progressive Renal Disease in Rats

**DOI:** 10.3390/ijms26199336

**Published:** 2025-09-24

**Authors:** Giampiero A. Massaro, Joana Mercado-Hernández, Roel Broekhuizen, Tri Q. Nguyen, Isabel Fuentes-Calvo, Sandra M. Sancho-Martínez, Carlos Martínez-Salgado, Francisco J. López-Hernández

**Affiliations:** 1 Instituto de Investigación Biomédica de Salamanca, 37007 Salamanca, Spain; giampieroandrea.massaro@usal.es (G.A.M.); joana.mercado.h@usal.es (J.M.-H.); ifc@usal.es (I.F.-C.); carlosms@usal.es (C.M.-S.); flopezher@usal.es (F.J.L.-H.); 2 Departamento de Fisiología y Farmacología, Universidad de Salamanca, 37007 Salamanca, Spain; 3 Group of Translational Research on Renal and Cardiovascular Diseases (TRECARD), 37007 Salamanca, Spain; 4 Department of Pathology, University Medical Centre Utrecht, 3584 Utrecht, The Netherlands; r.broekhuizen@umcutrecht.nl (R.B.);; 5 National Network for Kidney Research REDINREN, RICORS2040 RD21/0005/0004, Instituto de Salud Carlos III, 28029 Madrid, Spain; 6 Group of Biomedical Research on Critical Care (BioCritic), 47005 Valladolid, Spain

**Keywords:** acute kidney injury, chronic kidney disease, AKI-to-CKD transition, fibrosis, glomerular filtration, albuminuria

## Abstract

Chronic kidney disease (CKD) poses a global burden affecting over 10% of the adult population worldwide. Acute kidney injury (AKI) is an important cause of CKD, especially following severe and repeated episodes. However, the processes underpinning progressive and chronic renal deterioration after AKI are only incompletely understood. Thus, models reproducing this scenario are needed to study the pathophysiological mechanisms involved and identify biomarkers and molecular targets for diagnostic and therapeutic purposes. In this study, we developed a rat model of 3 serial AKIs leading to CKD, in which renal function, kidney structure and fibrosis, and urinary injury biomarkers were studied over a period of 9 months, alongside a traditional model of CKD caused by renal mass reduction. Our results show that consecutive AKIs eventually develop key features of CKD including progressive fibrosis and albuminuria. Renal injury biomarkers neutrophil gelatinase-associated lipocalin (NGAL), kidney injury molecule 1 (KIM-1), and retinol binding protein 4 (RBP4) show distinct evolution patterns suggestive of specific but undetermined damages with different time courses. The chronic evolution of renal tissue degeneration and dysfunction following serial AKIs closely resembles those observed after extensive renal mass reduction, which indicates chronic degeneration. Finally, a clear dissociation in the evolution of interstitial fibrosis (progressively increasing) and of glomerular filtration (mainly stable) was observed in both models. This questions the consuetudinary paradigm ascribing an etiological role to fibrosis in progressive renal dysfunction.

## 1. Introduction

Chronic kidney disease (CKD) constitutes a global health and socioeconomic burden with very high and growing prevalence worldwide [1]. It is estimated that CKD affects over 10% of the adult population, and that CKD will pose the second cause of death due to disease by 2100, after Alzheimer [2]. CKD is a multifactorial condition characterized by a chronic, progressive, and irreversible decline in overall renal excretory function associated with the pathognomonic loss of nephrons, tissue fibrosis and scarring, and impairment of glomerular filtration [3,4,5]. According to the Kidney Disease Improving Global Guidelines (KDIGO) Clinical Practice Guidelines, the decrease in the glomerular filtration rate (GFR) is usually preceded and subsequently accompanied by increasing albuminuria, which pose diagnostic hallmarks of this disease [6].

CKD patients face increased all-cause and cardiovascular mortality and reduced life expectancy [7] and, in end stage, become critically dependent on kidney transplant or dialysis. Genetic, toxic, autoimmune, infectious, and other conditions, but especially diabetes and hypertension pose the main causes of CKD and renal replacement therapies (RRT) [8]. Over the last decade, epidemiological and clinical studies have unveiled acute kidney injury (AKI), an abrupt decline in renal excretory function [9], as a risk factor for CKD [10,11,12]. Cumulative pathophysiological evidence also suggests a causal link between AKI and CKD [13,14].

Renal tubules show a remarkable but limited capacity for repair. Normally, following AKI, proximal tubules regenerate but only to a shorter length, with repeated AKI episodes accumulating further reductions [15]. Yet, especially after severe and repeated injuries, repair may also turn maladaptive and lead to CKD [16]. Maladaptive repair has been attributed to cell cycle stagnation and defective proliferation of regenerating cells leading to a secretory phenotype among proximal tubules within damaged nephrons and to characteristic features of chronic renal pathology such as interstitial inflammation and fibrosis [17,18,19].

Animal models are key to understanding the pathophysiological mechanisms of the transition from AKI to CKD and to developing diagnostic and therapeutic strategies. In fact, this transition, an otherwise silent stage, provides an opportunity for prophylactic intervention. However, existing models do not replicate human pathology with sufficient accuracy [16]. Beyond the artifactual nature of some experimental maneuvers (i.e., contralateral nephrectomy), present models are afflicted by the absence of progression [20,21,22,23,24,25], and mostly show tapering sequelae [20,21] or stable chronification [26]. Scarcely a few models develop a faint progression, only over a short period and at the expense of forcing experimental conditions [27,28]. Models indefinitely progressing over the long term are necessary to more closely mimic AKI-derived CKD.

In the present work we aimed to develop and report a rat model in which a serial combination of common acute injuries results in progressive renal tissue degeneration and albuminuria that are akin to those observed during the development of progressive CKD. Our model combines toxic (as in [20,21]) followed by successive bilateral ischemic (as in [28]) insults to add the cumulative effect of different pathological mechanisms. Homologous scenarios may be often found in the hospital setting or inadvertently in the community, where successive acute damage episodes of heterogeneous etiology may stochastically impact patients.

## 2. Results

### 2.1. Renal Structure Progressively Deteriorates over Time Following Triple AKI and RMR

**Figure 1:**
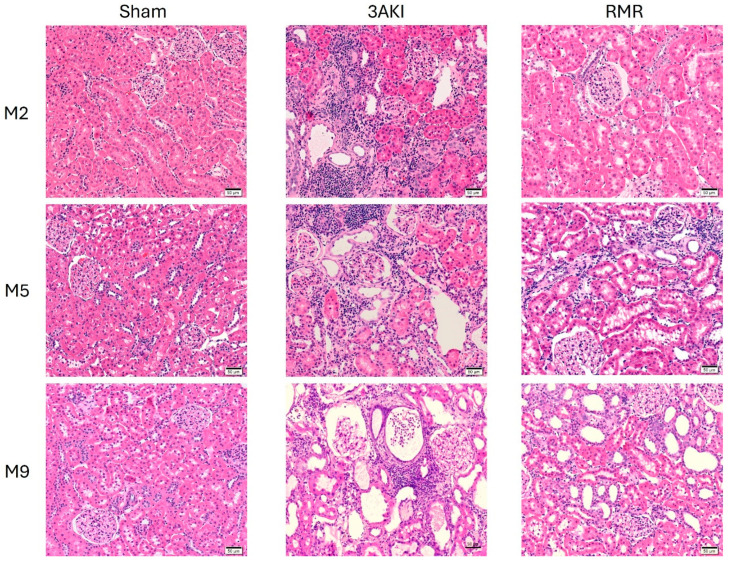
Representative images of the renal cortex of specimens from Control (Sham), 3AKI, and RMR groups (*n* = 3) stained with hematoxylin and eosin. M, month.

**Figure 2:**
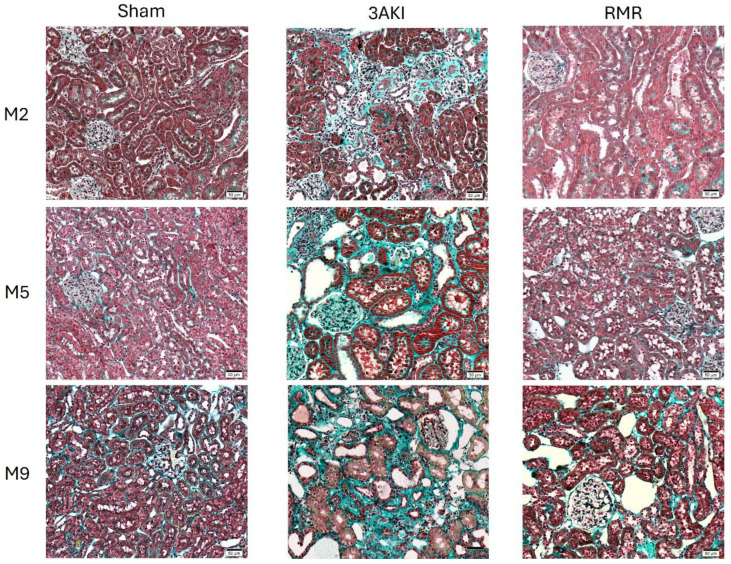
Representative images of the renal cortex of specimens from Control (Sham), 3AKI, and RMR groups (*n* = 3) stained with Mason’s trichrome. M, month.

**Figure 3:**
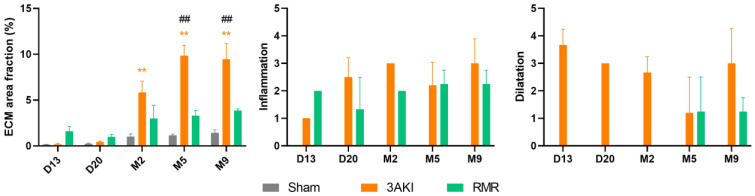
Evolution of renal tissue damage in Control (Sham), 3AKI, and RMR groups according to the level of fibrosis, tubular dilation, and inflammation. Data are expressed as the mean ± SEM of *n* = 3 per group. **, *p* < 0.01 versus Sham. ##, *p* < 0.01 versus RMR. AKI, acute kidney injury. ECM, extracellular matrix. RMR, renal mass reduction. M, month.

Hematoxylin-eosin staining of the renal cortex (Figure 1) and medulla (Supplementary Figure S1), Masson’s trichrome (Figure 2 and Supplementary Figure S2), and damage scorings, including automated quantification of the percentage of fibrotic areas, obtained from these specimens (Figure 3) show a progressive deterioration of the renal parenchyma in both models. However, as expected from the different etiopathology, some distinct features characterize their damage. In the 3AKI model, early time points are marked by acute injury as shown by more tubular dilatation, followed by increased inflammation and interstitial fibrosis at later time points. In the RMR model, no acute tubular dilation was observed at the early time points, as can be expected from the lack of acute tubular injury in this model. The tubular dilation observed at later time points is associated with increased interstitial fibrosis and inflammation. Notably, the development of interstitial fibrosis in the 3AKI model was more severe than in the RMR model.

### 2.2. Albuminuria and Transferrinuria Progressively Increase Following Triple AKI and RMR

**Figure 4:**
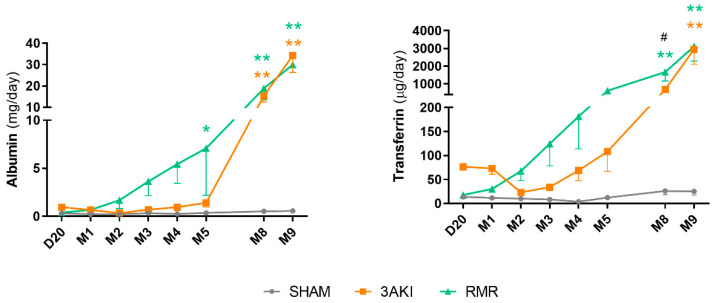
Evolution of albuminuria and transferrinuria in Control (Sham), 3AKI, and RMR groups. Experiments include *n* = 12 animals per group on D20. 3 animals per group were sacrificed on M2, 5 on M5, and 4 on M9. Data are expressed as the mean ± SEM. *, *p* < 0.05 versus Sham. **, *p* < 0.01 versus Sham. #, *p* < 0.05 versus RMR. AKI, acute kidney injury. RMR, renal mass reduction. D, day, M, month.

Renal tissue deterioration is also accompanied in both models by some functional alterations typical of CKD. As depicted in Figure 4, albuminuria and transferrinuria progressively increase in 3AKI and RMR rats, although at a different pace. Whereas albuminuria shows a steadier increment in RMR from early time points, its progression in 3AKI is notably slower in the first months and experiences an abrupt upward inflection in later stages. Similar findings were obtained for transferrinuria. In this case, however, its progression in 3AKI was delayed compared to RMR, but was steadier than that of albuminuria. In the later stages, both parameters reached very similar levels in both models. The biological and diagnostic meaning of albuminuria and transferrinuria seems very similar, but the different time course of both parameters in the two models reflects distinct damage evolution features.

### 2.3. Glomerular Filtration Remains Almost Stably Reduced in the Long-Term Following Triple AKI and RMR

**Figure 5:**
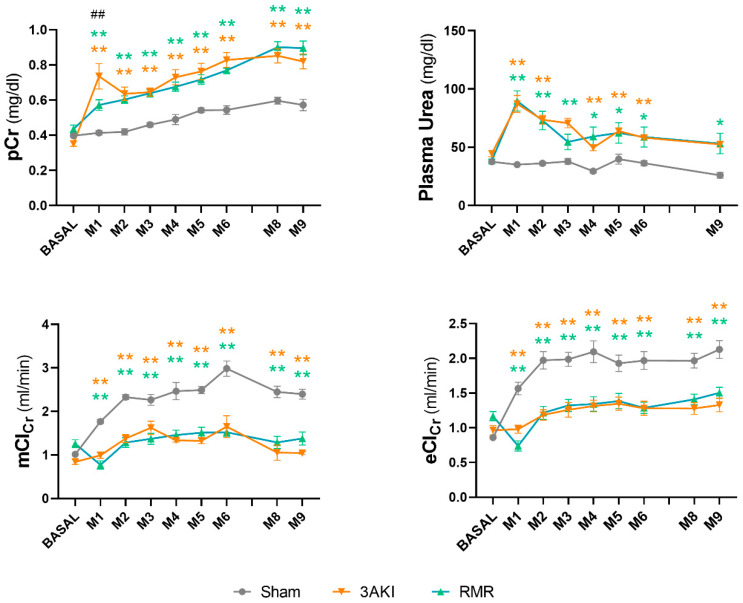
Evolution of glomerular filtration in Control (Sham), 3AKI, and RMR groups according to plasma creatinine concentration (pCr), plasma urea concentration, creatinine clearance (ClCr), and estimated ClCr using the ACLARA calculator. Experiments include *n* = 18 animals per group at basal timepoint, 12 at M1 and M2, 9 at M3-M5, 4 at M6-M9. Data are expressed as the mean ± SEM. *, *p* < 0.05 versus Sham. **, *p* < 0.01 versus Sham. ##, *p* < 0.01 versus RMR. AKI, acute kidney injury. D, day. eCl_Cr_, estimated creatinine clearance using ACLARA calculator. M, month. mCl_Cr_, measure creatinine clearance. pCr, plasma creatinine concentration. RMR, renal mass reduction.

Interestingly, the evolution of the filtration function, as evidenced by measured and estimated GFR, pCr and plasma urea concentration (Figure 5), reflects an impairment caused by the initial aggression inflicted in both models (i.e., repeated AKIs in the 3AKI, and abrupt renal mass dissection in the RMR), with no significant progressive decline observed thereafter. For the record, the evolution of kidney function through the acute period is shown in Supplementary Figure S3.

### 2.4. Urinary Injury Biomarkers Show Particular Trends Following Triple AKI and RMR

**Figure 6:**
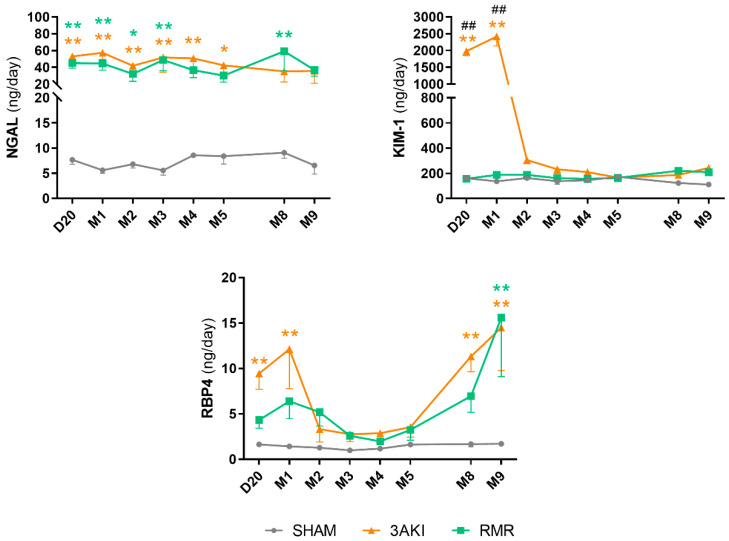
Evolution of injury biomarkers NGAL, KIM-1, and RBP4 in Control (Sham), 3AKI, and RMR groups. Experiments include *n* = 12 animals per group on D20. 3 animals per group were sacrificed on M2, 5 on M5, and 4 on M9. Data are expressed as the mean ± SEM. *, *p* < 0.05 vs. Sham. **, *p* < 0.01 vs. Sham. ##, *p* < 0.01 vs. RMR. AKI, acute kidney injury. RMR, renal mass reduction. D, day, M, month.

The so-called urinary *injury biomarkers* are gaining increasing attention and erupting into the clinical scenario, for their association with renal injury independently, and even in the absence, of increments in pCr. These biomarkers typically include KIM-1, NGAL, and RBP4 [29,30], which are sensitive indicators of renal tubular damage. Figure 6 shows that the evolution of these biomarkers is distinct and particular for each one and, in general terms, very similar in both models beyond the differences arising during the different acute phases (i.e., the three consecutive AKIs in the 3AKI, and the abrupt renal mass reduction in the RMR). Different biomarkers provide complementary pathophysiological information potentially affording enhanced diagnostic granularity on different aspects of tubular damage that needs to be further investigated. KIM-1 shows a profile consistent with a marker of lingering sequelae of AKI, as previously shown [21]. The tendency followed by RBP4 is very coincident with that of transferrin, and the stably increased level of urinary NGAL excretion might reflect the level of active renal damage of yet-unspecified nature, as suggested [31], but similarly common to both models. This level of damage may be reflected by the level of inflammation, which has been related to NGAL renal production [32].

## 3. Discussion

Overall, the model of three consecutive acute damages induces a subsequent scenario of progressive and irreversible deterioration of renal tissue structure and function, very similar to that observed after extensive renal mass reduction but with distinctive features. Indeed, tissue degeneration and fibrosis continuously increase over time along with albuminuria and transferrinuria in both models, although to a higher degree in the 3AKI. Despite almost completely recovered after the acute phase, the initial concatenation of acute damages of different etiopathology seeds the grounds for a worse chronic evolution than renal mass reduction. In contrast, glomerular filtration shows a chronically stable impairment inflicted by the initial aggression (i.e., serial AKIs or abrupt RMR), but its long-term evolution is nearly identical to that observed in healthy rats in the control group. Serial AKIs may lead the kidneys to an apparently full recovery, clinically indistinguishable from normal in most cases in practice. Nonetheless, our model shows that this apparent normality hides maladaptive sequelae that eventually cross a point of no return towards progressive and irreversible deterioration that becomes independent of additional insults.

These findings reinforce the role of AKI in the etiopathology of CKD and contribute to supporting the need of reconsidering the importance of AKI on global health [33,34]. In fact, AKI is quite incident in the hospital setting (2–7%), and bolder numbers might be concealed in the community [11,35]. Only hospital admissions related to community-acquired AKI double hospital-acquired AKI cases [36,37], but the total incidence of community-acquired AKI remains uncertain because reports are mostly limited to patients admitted to hospitals [35]. Even if less severe cases were those passing unnoticed in the community, mild and subclinical AKI episodes have been shown to impact morbidity and mortality in the medium and long terms [10,38,39,40]. Our results are consistent with these facts. In our 3AKI model, the second insult (i.e., left renal ischemia) contributing to the subsequent chronic development causes a mild AKI that would otherwise pass unnoticed if not experimentally monitored (Supplementary Figure S3), resembling a prototypical case plausibly occurring in the community. Furthermore, in most instances the transition from resolved AKI to CKD courses clinically silent and blind to present diagnostic technology [13]. Thus, new tools are necessary for routine screening within the community that ideally identify patients before they cross the no-return point. Beyond this threshold, diagnosis turns into early CKD recognition, and prospective prophylaxis into early treatment.

An unmet key to this aim is the differentiation of AKI sequelae and pathophysiological mechanisms that eventually taper off, from those that corrupt the renal environment towards irreversible progression. The term maladaptive repair defines a scenario of atypical restoration from AKI in which the renal parenchyma does not fully recover its original structure, and inflammation and fibrosis, two related pathognomonic processes traditionally associated to chronification, install [17,41]. The causes and mechanisms driving renal repair to a maladaptive scenario are still undetermined, but a chief role for alterations in the timely ulterior switch off of the SRY-Box transcription factor 9 (SOX9) in regenerating renal cells has been recently implicated [42,43]. However, there is no compelling evidence that the inflammation and fibrosis resulting from maladaptive repair progress and spread over time. Furthermore, despite amply assumed by the nephrology community [44], it is incompletely understood whether inflammation and fibrosis are etiopathogenic factors promoting progression and degeneration or pose adaptive, response, or even merely attendant phenomena.

Renal fibrosis is an ambiguous term referring to the excessive deposition of extracellular matrix that may be found in sclerotic glomeruli, in the interstitium, around renal vessels, or forming part of the scar refilling the space left by lost nephrons. Renal scarring is the product of a wound healing-like process occurring in disease kidneys [45] that provides a structural scaffolding solution by replacing disintegrated tissue with fibrotic material [46]. Traditionally, fibrosis has been considered a driver of progressive tissue degeneration and organ dysfunction [41]. The impact of fibrosis is obvious in organs such as the heart and the lungs whose proper function critically depends on their distensibility and elasticity. However, this association is less intuitive for other organs, such as the kidneys. In fact, neither wound healing in general [47] nor renal wound healing in particular associate with further or progressive tissue degeneration. For instance, wounds and incisions made in the kidneys during nephrotomies and renal tumor extirpations undergo normal healing [48,49] with no potential complications other than bleeding and infections [49,50], which indicates that fibrosis might not necessarily be a progression factor *per se*. In agreement, blockade of transforming growth factor beta 1 (TGFβ1), a major promoter of renal fibrosis [44,51,52], has failed to provide benefits on CKD in clinical studies [53].

The results of our study also show a disconnection of overall kidney fibrosis with certain aspects of renal dysfunction in both models, mainly with glomerular filtration, the signature feature of CKD. Indeed, if fibrosis were a progression factor contributing to organ dysfunction, it would be difficult to explain how, in both models, the filtration function evolves in the long term almost identically to that in the control group in a scenario of rampantly increasing fibrosis (more accentuated in 3AKI). This dissociation suggests that further granularity is necessary to analyze the impact of fibrosis on disease evolution. Likely, the pathophysiological effect of the fibrotic processes occurring in different renal compartments (i.e., glomerular, interstitial, perivascular, and scar), as well as their time course, distinctly influence CKD progression. From a merely speculative perspective, whereas the fibrotic material forming part of the refilling scarring may have a more inert role imposed by its spatial delimitation, interstitial fibrosis directly connected and intertwined with capillary and tubular structures might affect their function and promote a vicious cycle of degeneration [4,5]. Different mechanisms have been proposed, including capillary rarefaction and tubular damage and activation derived from the interference of fibrotic materials with adhesion and cell-to-cell communication integrins and other membrane components (e.g., release of nested TGFβ), contributing to generating hypoxic and pro-inflammatory environments [44,51,52].

Moreover, the etiopathological relation among fibrosis, albuminuria, and GFR is still unclear and, probably, model specific, and must be analyzed also with this scope. The potential relation of fibrosis with albuminuria (and transferrinuria) is even more complex than with glomerular filtration. Increased albuminuria may result from abnormally elevated filtration secondary to derangement of the glomerular filtration barrier (GFB) overwhelming the tubular reabsorption capacity, or from defective tubular reclamation (or a mixture of both). Compartmental fibrosis is expected to relate differently to albuminuria depending on the initiating injury mechanism and damage pattern. This is evident in the different courses of albuminuria (and transferrinuria) and fibrosis in both models. It can be hypothesized that, in the RMR model, albuminuria and transferrinuria could be the consequence of its increased filtration (as renal mass reduction forces extant nephrons to increase filtration, which eventually leads to glomerular and GFB damage). In the 3AKI model, on the contrary, because cisplatin and ischemia mostly damage the tubular compartment, it is more reasonable to speculate that albuminuria and transferrinuria arise from defective tubular reuptake. As a corollary, glomerular and tubular fibrosis may distinctly modulate protein excretion and, thus, their biological and diagnostic meaning is also context specific.

The pattern of urinary biomarkers is also model and biomarker specific. As argued elsewhere [54], the specific and individual biological meaning of the increased urinary excretion of the so-called injury biomarkers is far from understood. Biomarkers long thought to be directly shed to the evolving urine by damaged tubules, such as NGAL, TIMP-1 and IGFBP7, have been shown to also reflect impaired tubular reuptake in certain scenarios [55,56,57]. Moreover, urinary injury biomarkers do not necessarily correlate with the extent of renal damage [58], nor do they behave in unison [54,59]. In fact, patients and animal models suffering renal damage may commonly be positive to a specific biomarker and negative to another. This indicates that the bodily traffic of each individual biomarker responds to a particular combination of injury to different renal structures and alterations in their renal handling resulting in increased appearance in the urine. Consequently, much more investigation is needed to unveil the precise pathophysiological information provided by individual biomarkers beyond the existence of undetermined damage.

Thus, we can only speculate about the meaning of NGAL, KIM-1 and RBP4 in our models. Interestingly, KIM-1 has been associated with renal repair from AKI [21], which is congruent with the evolution patterns observed in the 3AKI and RMR animals. In the former, acute damage to renal structures (i.e., the tubules) initially elevated urinary KIM-1, which tapers off in parallel to renal repair, as we have demonstrated previously [21]. RMR animals, on the contrary, did not suffer acute damage to renal structures, and thus their urinary KIM-1 remained normal through the experiment. NGAL has been associated with AKI but also to renal and systemic inflammatory syndromes and acute response. As shown in Figure 4, both models show a considerable and rather stable level of renal inflammation through the experiment, coinciding with the elevated urinary excretion of NGAL. Finally, RBP4 is a very sensitive marker of tubule (especially proximal tubule) alterations [30]. Its urinary excretion increases as a result of defective reuptake caused by tubular damage or the reabsorptive capacity of the proximal tubules is exceeded. The evolution of urinary RBP4 is very similar in both models. The early peak observed by month one may be reflective of tubular damage in the case of 3AKI animals, and of overwhelmed tubular reclamation in RMR rats. In fact, extant nephrons are forced to markedly increase single nephron GFR to compensate for the abrupt decline in filtration resulting from extensive renal mass ablation. It may be hypothesized that, in this scenario, the maximal reabsorption is exceeded temporarily until eventually readjusted. Its new increase in advanced stages of progressive renal degeneration may reflect tubular performance impairment. Altogether, more research is necessary to fully understand the contextualized and collective meaning of individual urinary biomarkers, to an extent useful for clinical diagnostic application.

In summary, the triple AKI model recapitulates some of the main characteristics of the transition to a truly progressive and irreversible chronic kidney disease, which reinforces the etiopathological role of AKI on CKD, and provides a good experimental tool to peruse this condition at the pathophysiological, diagnostic, prophylactic, and therapeutic levels. Moreover, our study also suggests that the etiopathological interplay of structural and functional alterations in CKD should be reconsidered under more detailed and context-specific information. Although most forms of CKD share a common final phenotype, their initiation and early development stages, where irreversibility occurs and intervention is envisaged, are different and differentially represented by standard and new biomarkers.

## 4. Materials and Methods

Unless otherwise indicated, reagents were purchased from Merck (Madrid, Spain).

### 4.1. Animal Model and Experimental Protocol

Animal studies were performed in accordance with the Principles of the Declaration of Helsinki, following the European Guide for the Care and Use of Laboratory Animals (Directive 2010/63/UE) and the Spanish national and regional regulations (Law 32/2007/Spain, RD 1201/2005 and RD 53/2013). All the procedures were approved by the Bioethics Committee for Animal Care and Use of the University of Salamanca (Salamanca, Spain).

**Figure 7:**
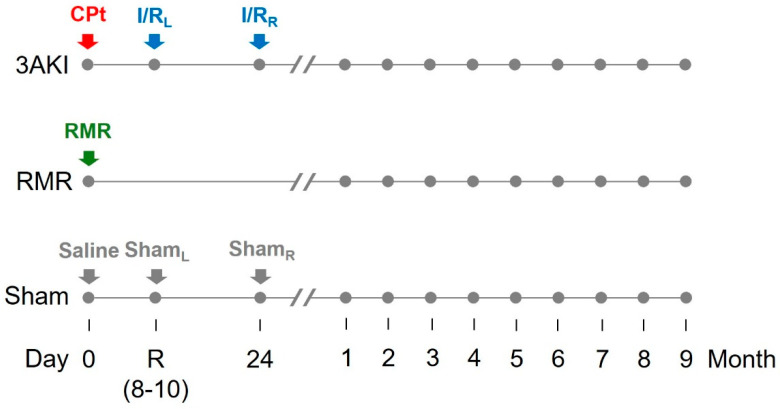
Graphical depiction of the experimental protocol and timeline. AKI, acute kidney injury. CPt, cisplatin. I/R, ischemia/reperfusion. L, left. R, right. RMR, renal mass reduction.

Male Wistar rats (200–230 g) were housed under controlled environmental conditions, fed with standard chow, and allowed free access to drinking water. Rats were subjected to Triple AKI (as described above), or renal mass reduction (RMR, a classical model of chronic renal damage, for comparison), or to sham procedures (as control), and were followed for 9 months (Figure 7). For this purpose, rats were randomly divided into the following groups:

- Triple AKI group (3AKI): rats received a single i.p. dose of cisplatin (5 mg/kg) on day 0 (basal), and were subsequently subjected to a 60-min ischemia by placing a clamp in the renal artery of the left kidney on day R (i.e., recovery from first AKI as by plasma creatinine concentration (pCr) normalization, typically 7–10 days after the insult), and to a 60-min ischemia by placing a clamp in the renal artery of the right kidney on the day 24. Renal ischemia/reperfusion was carried out basically as described [60]. Briefly, rats were anesthetized with a mixture of 80 mg/kg ketamine and 20 mg/kg xylazine. Kidneys were accessed through a medial laparotomy and the renal artery gently exposed, cleaned from surrounding fat, and clamped for 60 min, after which the clamp was removed to allow reperfusion. Incisions were then sutured, and animals were allowed to recover under 30 μg/kg buprenorphine analgesia (every 12 h for two days).
- Renal mass reduction (RMR) group: rats were subjected to renal mass reduction at day 0 (basal). Renal mass reduction was achieved by right nephrectomy and polectomy of the left kidney, as described [61]. Briefly, rats were anesthetized with a mixture of 80 mg/kg ketamine and 20 mg/kg xylazine. Kidneys were then accessed through a medial laparotomy. The right kidney pedicle was ligated, and the kidney was dissected. The poles of the left kidney were severed with a scalpel and absorbable hemostatic tissue (Espongostan® Film, Takeda, Madrid, Spain) was applied to prevent bleeding. Animals were sutured and allowed to recover under 30 μg/kg buprenorphine analgesia (as above).
- Control group (sham): rats were administered physiological saline on day 0 (basal) and were subjected to sham-operations on day R and 24.

At designated time points, rats were allocated to metabolic cages to obtain individual, 24-h urine and blood samples. Urine was cleared by centrifugation and stored at −80 °C. At those same times, 200 μL blood samples were obtained with a needle placed in the tail vein and immediately centrifuged to obtain the plasma, which was stored at –80 °C. At specific time points, some animals were anesthetized with 50 mg/kg sodium pentobarbital and their kidneys were perfused with saline solution through the aorta to remove the blood, dissected, and fixed in 3.7% paraformaldehyde. Then, animals were sacrificed by exsanguination.

### 4.2. Renal Histology

Fixed kidney specimens were embedded in paraffin, cut into 3 μm thick slices, and stained with hematoxylin and eosin (HE) and Masson’s Trichrome (MT). Microphotographs were obtained using the DotSlide virtual microscopy technique (Olympus BX51, Olympus Iberia, Barcelona, Spain). Image analysis was performed with Olyvia v2.6 Software (Olympus Iberia). The severity of renal damage was assessed blindly by an expert renal pathologist. Signs of tubular injury (dilatation) and inflammation were assessed on HE- and TM-stained slides and injury was graded on a scale from 0–4.

The severity of interstitial fibrosis was assessed by morphometry. For this, MT-stained slides were scanned with the NanoZoomer S360 scanner (Hamamatsu Photonics, Hamamatsu City, Japan) at 400x magnification. The scanned images were imported in QuPath v0.4.3 in a single project and the tissue areas were annotated using QuPath’s simple tissue detection and manually corrected. Representative regions were combined into a training image. With this training image a pixel classifier (Artificial neural network) was trained to identify the area that was stained by the MT-staining at high (0.45 µm) resolution. Special care was taken not to include the dark blue/green staining that was present in the brush border areas. Finally, the total area and positive area in each tissue section annotation were measured. Results are presented as % positive area.

### 4.3. Renal Function

pCr, urinary creatinine (uCr) and proteinuria were analysed using commercial kits based respectively on Jaffe’s reaction and Bradford method, following the manufacturer’s instructions (BioAssay System, Hayward, CA, USA). GFR was measured by the creatinine clearance (Cl_Cr_) method, according to the following formula:

Cl_Cr_ = uCr × UF/pCr, where UF is the urine flow.

Creatinine clearance was also estimated with the ACLARA calculator (https://idal.uv.es/aclara/, accessed on 14 January 2025), based on plasma creatine and body weight [62]. Plasma urea concentration was measured with the Urea Assay Kit (Biochain®, Newark, CA, USA) according to the manufacturer’s instructions.

### 4.4. Urinary Biomarkers

The following biomarkers were measured with commercial ELISAs according to the manufacturers’ instructions: neutrophil-associated gelatinase lipocalin (NGAL) with the Rat NGAL ELISA KIT 046 (Bioporto Diagnostics, Hellerup, Denmark); kidney injury molecule 1 (KIM-1) with the Rat Kidney Injury Molecule, KIM-1, ELISA kit CSB-E08808r (CUSABIO, Houston, TX, USA); albumin with the Rat Albumin ELISA Kit ab108789 (Abcam, Cambridge, UK); transferrin with the Rat Transferrin ELISA Kit ab137992 (Abcam, Cambridge, UK); and retinol binding protein 4 (RBP4) with the Rat Retinol Binding Protein 4 ELISA Kit ab203362 (Abcam, Cambridge, UK). Biomarker concentration values were referred to the corresponding uCr.

### 4.5. Statistical Analysis

Statistical analyses were performed using GraphPad Prism 7 software (San Diego, CA, USA). Data are expressed as the mean ± standard error of the mean (SEM). Data distribution was evaluated with the Shapiro–Wilk normality test. The two-way ANOVA followed by Tukey’s post hoc test was used to compare experimental groups over time. A *p* value < 0.05 was considered statistically significant.

## Data Availability

Data are available upon reasonable request.

## Supplementary Materials

The following supporting information can be downloaded at: https://www.mdpi.com/article/10.3390/ijms26199336/s1.

## Glossary

AKI	acute kidney injury
Cl_Cr_	creatinine clearance
CKD	chronic kidney disease
KIM-1	kidney injury molecule 1
GFB	glomerular filtration barrier
GFR	glomerular filtration rate
HE	hematoxylin and eosin
KDIGO	Kidney Disease Improving Global Clinical Practice Guidelines
MT	Masson’s Trichrome
NGAL	neutrophil gelatinase-associated lipocalin
pCr	plasma creatinine concentration
RBP4	retinol binding protein 4
RMR	renal mass reduction
RRT	renal replacement therapies
SEM	standard error of the mean
SOX9	SRY-Box transcription factor 9
TGF	transforming growth factor
uCr	urinary creatinine concentration
UF	urinary flow
