# Supplementary material for: Serial Combination of Toxic and Ischemic Renal Damages Causes Subsequent Chronic, Irreversible, and Progressive Renal Disease in Rats
Source: Int J Mol Sci. 2025 Sep 24;26(19):9336. doi: 10.3390/ijms26199336 (PMC12525075; doi:10.3390/ijms26199336)
Supplement: Supplementary file 1 [file ijms-26-09336-s001.zip › Captions_Supplementary S1-S3.docx]

**Supplementary Figure 1.** Representative images of the renal medulla of specimens from Control (Sham), 3AKI, and RMR groups (n=3) stained with hematoxylin and eosin. AKI, acute kidney injury. M, month. RMR, renal mass reduction.

**Supplementary Figure 2.** Representative images of the renal medulla of specimens from Control (Sham), 3AKI, and RMR groups (n=3) stained with Mason’s trichrome. AKI, acute kidney injury. M, month. RMR, renal mass reduction.

**Supplementary Figure 3.** Evolution of renal function in the 3AKI (n=14) and Sham (n=14) groups as informed by plasma creatinine concentration (pCr), plasma urea concentration, creatinine clearance (Cl_Cr_), and estimated Cl_Cr_ using the ACLARA calculator. Data are expressed as the mean ± SEM. *, p < 0.05 versus Sham. **, p < 0.01 versus Sham. D, day. M, month.
