# Supplementary figures and images for: Serial Combination of Toxic and Ischemic Renal Damages Causes Subsequent Chronic, Irreversible, and Progressive Renal Disease in Rats
Source: Int J Mol Sci. 2025 Sep 24;26(19):9336. doi: 10.3390/ijms26199336 (PMC12525075; doi:10.3390/ijms26199336)

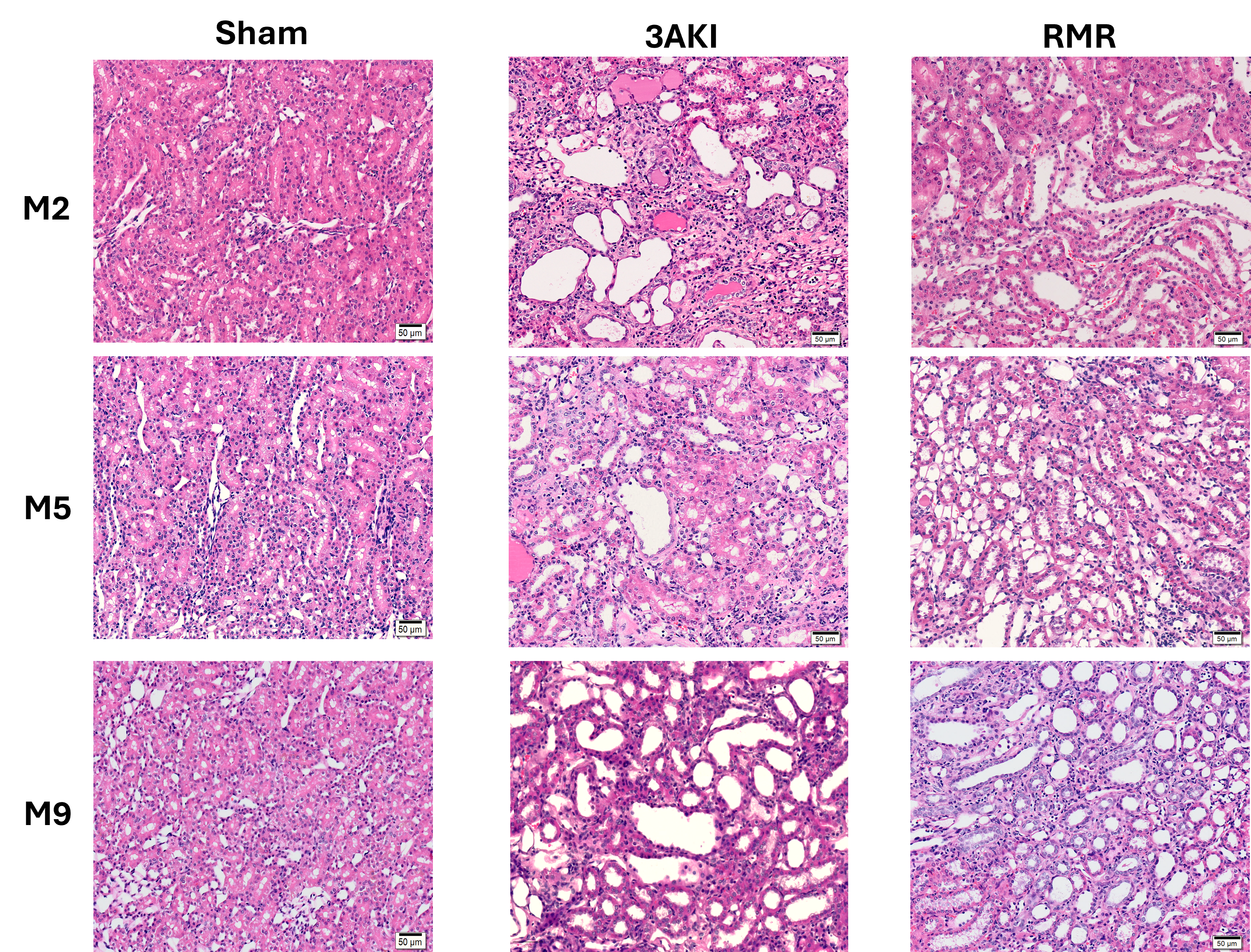

Supplement: Supplementary file 1 [file ijms-26-09336-s001.zip › sup. fig. 1.png]

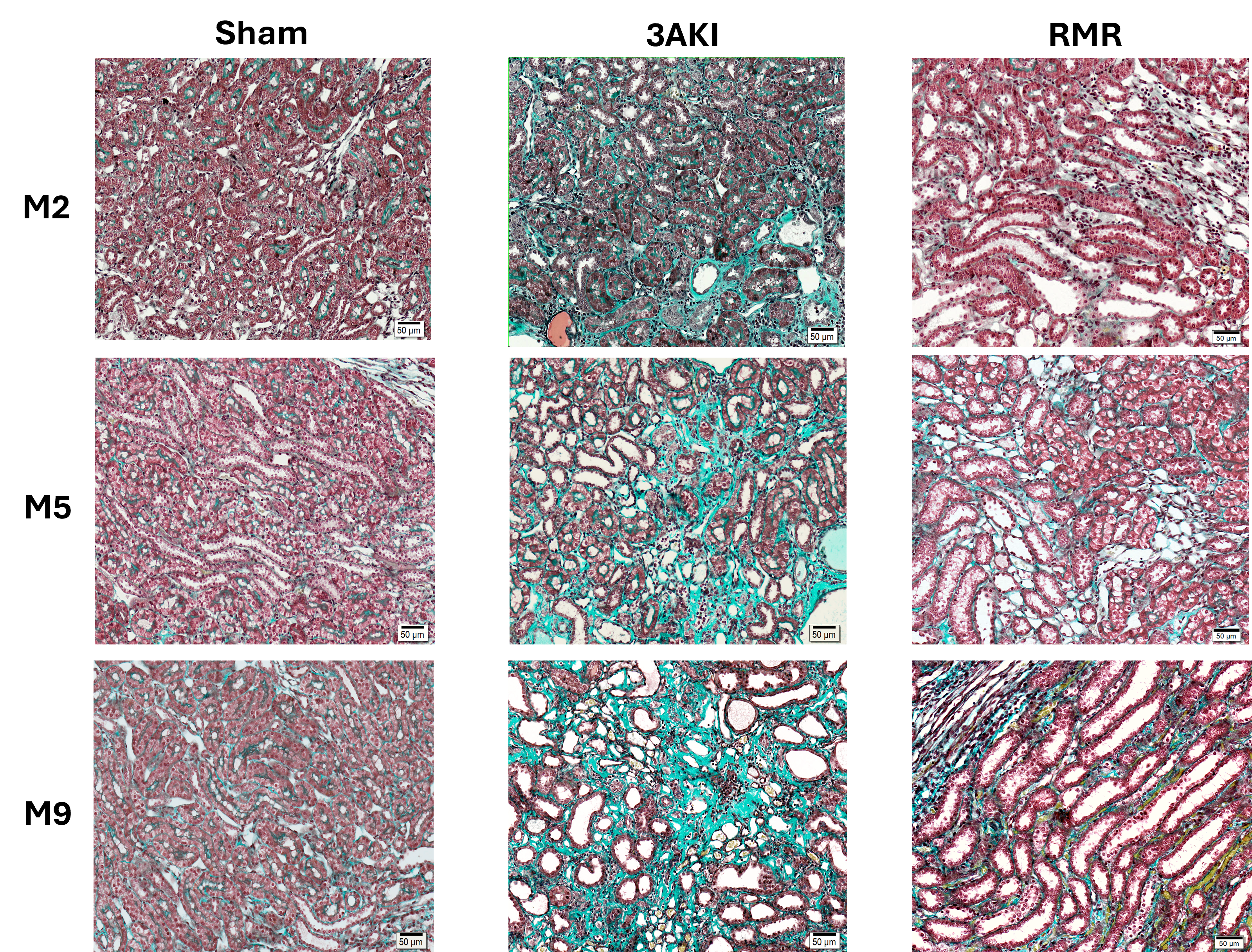

Supplement: Supplementary file 1 [file ijms-26-09336-s001.zip › sup. fig. 2.png]

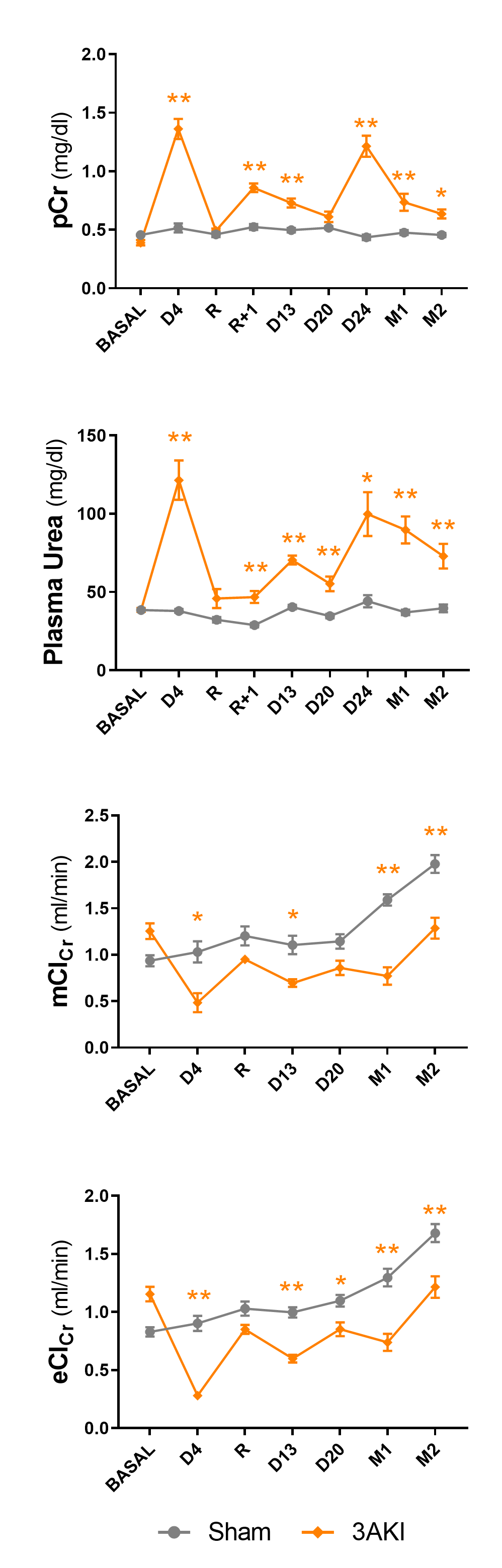

Supplement: Supplementary file 1 [file ijms-26-09336-s001.zip › sup.fig.3.tif]
